# Targeting SYK enhances responses to radiotherapy and PD-1 blockade with myeloid immune remodeling in breast cancer

**DOI:** 10.3389/fimmu.2026.1929236

**Published:** 2026-09-18

**Authors:** Yiqun Han, Qin Zhou, Anna Dibbs, Shouhai Zhu, Ping Yin, Kevin D. Pavelko, Haidong Dong, Kuntian Luo, Xinyi Tu, Fabrice Lucien-Matteoni, Sean S. Park, Michael P. Grams, Meng Xu-Welliver, Cameron M. Callaghan, Zhenkun Lou, Robert W. Mutter

**Affiliations:** 1 Department of Radiation Oncology, Mayo Clinic, Rochester, MN, United States; 2 Department of Oncology, Mayo Clinic, Rochester, MN, United States; 3 Department of Immunology, Mayo Clinic, Rochester, MN, United States; 4 Department of Urology, Mayo Clinic, Rochester, MN, United States; 5 Department of Molecular Pharmacology and Experimental Therapeutics, Mayo Clinic, Rochester, MN, United States

**Keywords:** myeloid reprogramming, radiation therapy, spleen tyrosine kinase, triple-negative breast cancer, tumor microenvironment

## Abstract

**Introduction:**

Spleen tyrosine kinase (SYK) is a non-receptor tyrosine kinase involved in immune signaling and has been implicated in tumor-intrinsic resistance to DNA-damaging therapies. Although SYK activity has been associated with poor outcomes in triple-negative breast cancer (TNBC), its role in resistance to combined radiotherapy and immune checkpoint blockade remains unclear.

**Methods:**

SYK expression and clinical relevance were assessed across multiple breast cancer cohorts, including radiotherapy-treated datasets. Integrated transcriptomic analyses were used to characterize SYK-associated immune states. The functional effects of pharmacologic SYK inhibition were evaluated using *in vitro* assays and an immunocompetent bilateral 4T1 tumor model in female BALB/c mice treated with radiotherapy and PD-1 blockade. Treatment-associated immune remodeling was assessed by flow cytometry and transcriptomic profiling.

**Results:**

Across multiple cohorts, elevated SYK expression, particularly in TNBC, was associated with inferior clinical outcomes and immune dysregulation. Pharmacologic SYK inhibition enhanced tumor responses to radiotherapy and PD-1 blockade at both irradiated and distant tumor sites. These effects were accompanied by coordinated remodeling of the tumor immune microenvironment, including suppression of neutrophil-associated signatures and reductions in immunosuppressive myeloid populations, including M2-like macrophages and myeloid-derived suppressor cells.

**Discussion:**

Pharmacologic SYK inhibition enhances responses to radiotherapy and PD-1 blockade in preclinical TNBC models and is accompanied by remodeling of the immunosuppressive myeloid compartment. These findings support further investigation of SYK-targeted strategies to improve responses to combined radiotherapy and immune checkpoint blockade.

## 1 Introduction

Spleen tyrosine kinase (SYK) is a cytoplasmic non-receptor tyrosine kinase that transduces signals from immunoreceptors, integrins, and Fc receptors, thereby regulating diverse cellular processes including proliferation, survival, and cytokine release (1). While SYK has been most extensively studied in B-cell receptor signaling and lymphoid malignancies, accumulating evidence indicates that it also plays important roles in solid tumors (2–5). *SYK* expression is upregulated in breast, colorectal, pancreatic, and lung cancers, where it has been reported to exert context-dependent effects ranging from tumor-promoting to tumor-suppressive activity (6–9). Beyond direct effects on tumor cells, SYK has been implicated in myeloid cell regulation and inflammatory signaling, suggesting that it may act as a critical interface between tumor-intrinsic biology and the tumor immune microenvironment (10–12). In particular, SYK has emerged as an important regulator of macrophage biology, influencing Fc receptor-dependent phagocytosis, cytokine secretion, and polarization (1, 13).

Triple-negative breast cancer (TNBC) is an aggressive and therapy-resistant breast cancer subtype defined by the lack of expression of the estrogen receptor (ER), progesterone receptor (PR), and the human epidermal growth factor receptor 2 (HER2) gene. TNBC accounts for approximately 15% of all breast cancer cases, and is characterized by early relapse, high rates of metastasis, and poor survival (14). Adjuvant radiotherapy is the standard of care in all patients with early-stage TNBC following breast conserving surgery and is strongly considered in patients with lymph node positive disease treated with mastectomy (15). The incorporation of pembrolizumab into neoadjuvant chemotherapy, followed by adjuvant pembrolizumab and radiotherapy as clinically indicated has improved outcomes in patients with early-stage TNBC; however, therapeutic resistance remains a major clinical challenge as patients residual disease after neoadjuvant systemic therapy remain at elevated risk of relapse (16, 17).

Radiotherapy has both immune activating and immunosuppressive effects (18). While radiation can stimulate immune recognition through the release of tumor antigens and danger signals, this response is frequently blunted by the presence of immunosuppressive myeloid cells, including immunosuppressive M2-like tumor-associated macrophages (TAMs) and myeloid-derived suppressor cells (MDSCs). These populations promote immune evasion, foster tumor progression, and represent a major barrier to disease control (19). There is a pressing need to develop new combinations to enhance the efficacy of radiotherapy plus PD-1 inhibition, particularly in patients with residual disease after neoadjuvant systemic therapy who are at high risk of relapse and death. Given the established role of SYK in myeloid-cell signaling and macrophage polarization (12, 20), we hypothesized that SYK inhibition could complement radiotherapy and PD-1 blockade by simultaneously targeting tumor-intrinsic radioresistance and immunosuppressive myeloid remodeling.

Recent data indicates that the successful integration of radiotherapy with PD-1/PD-L1 inhibition requires remodeling of the tumor immune microenvironment (21). Macrophages are central in this process, as they can adopt either a pro-inflammatory (M1-like) or immunosuppressive M2-like phenotypes, thereby shaping T cell infiltration and function (22, 23).

In this study, we comprehensively investigated the function of SYK in TNBC by integrating transcriptomic datasets, *in vitro* mechanistic assays, and *in vivo* models. We showed that *SYK* is highly expressed in TNBC and is associated with immune exhaustion and poor outcomes in patients treated with radiotherapy. Pharmacologic inhibition of SYK sensitizes TNBC cells to radiotherapy and DNA-damaging agents, remodels the tumor immune microenvironment with prominent macrophage reprogramming, and enhances abscopal response to radiotherapy combined with PD-1 blockade. Together, these findings identify SYK as a mediator of radioresistance and immune dysfunction in TNBC and highlight its inhibition as a promising therapeutic strategy to improve the efficacy of adjuvant therapy.

## 2 Methods

### 2.1 Bulk RNA-sequencing and analysis

Tumor samples were harvested from bilateral 4T1-bearing mice at predefined timepoints following treatment with vehicle (Ctrl), RT plus anti-PD-1 antibody (RP), or the triple combination of RT, anti-PD-1 antibody, and SYK inhibitor (SYKi) fostamatinib (R788; referred to as SYKi throughout the experimental figures) (RPS). Tumors were bisected longitudinally; one portion was snap-frozen in liquid nitrogen for RNA extraction. Total RNA was isolated using the RNeasy Mini Kit (Qiagen), including on-column DNase I treatment to eliminate genomic DNA. RNA integrity was confirmed using an Agilent 2100 Bioanalyzer, and only samples with RNA integrity number (RIN) ≥ 7.0 proceeded to library construction. RNA-seq libraries were generated using the TruSeq Stranded mRNA Library Prep Kit (Illumina) according to the manufacturer’s guidelines. Libraries were quantified using Qubit (Thermo Fisher Scientific), and size distribution was confirmed using a Bioanalyzer. Sequencing was performed on an Illumina NovaSeq 6000 platform with paired-end 150 bp reads, targeting a depth of approximately 30 million reads per sample.

Raw sequencing reads were trimmed for quality and adapters using Trimmomatic and aligned to the mouse reference genome (GRCm38/mm10) using STAR. Gene-level quantification was performed using featureCounts, and raw count matrices were imported into R for downstream processing. Differential gene expression analysis was conducted using the DESeq2 package. Genes with a Benjamini-Hochberg adjusted p-value (FDR) < 0.05 and absolute log2 fold change > 1 were considered significantly differentially expressed. Gene Ontology (GO) and Kyoto Encyclopedia of Genes and Genomes (KEGG) pathway enrichment analyses were performed using Metascape database (https://metascape.org) or the clusterProfiler package in R. Enrichment results were ranked based on adjusted p-values and visualized using dot plots and bar graphs. Single-sample gene set enrichment analysis (ssGSEA) was performed using the GSVA package; normalized enrichment scores (NES) were calculated for each sample, and treatment group comparisons were made using nonparametric statistical testing. Tumor-infiltrating immune cell populations were inferred from bulk RNA-seq data using the TIMER2.0 platform. Computationally inferred immune-cell abundance was compared across treatment groups. RNA-seq analyses were performed using three independent tumors per treatment group (n = 3), with each tumor considered an independent biological replicate.

### 2.2 CyTOF profiling of the tumor microenvironment

Tumors were harvested from bilateral 4T1-bearing mice on days 7 and 14 following treatment with Ctrl, RP, or RPS. Tumors were mechanically dissociated and enzymatically digested in RPMI-1640 medium containing 1 mg/mL collagenase type IV (Sigma), 0.1 mg/mL DNase I (Roche), and 2% fetal bovine serum (FBS) at 37 °C for 30 minutes. Cell suspensions were filtered through 70-µm nylon strainers, followed by red blood cell lysis using ACK lysis buffer. Viable single-cell suspensions were counted and processed immediately for CyTOF staining.

Cells were incubated with 5 µM Cell-ID Cisplatin (Fluidigm) for 5 minutes at room temperature to label dead cells, followed by quenching and washing. After Fc receptor blocking with anti-mouse CD16/32 (BioLegend), cells were stained with a pre-titrated cocktail of metal-conjugated antibodies targeting surface markers for 30 minutes at 4 °C. Cells were then fixed and permeabilized using the Foxp3 Transcription Factor Staining Buffer Set (Thermo Fisher Scientific) according to the manufacturer’s instructions, followed by intracellular staining with antibodies against transcription factors and cytokines for 45 minutes. Cells were subsequently fixed overnight in 1.6% paraformaldehyde containing Cell-ID Intercalator-Ir (Fluidigm) for DNA labeling. Prior to acquisition, cells were washed and resuspended in EQ Four Element Calibration Beads (Fluidigm) diluted in Maxpar Cell Acquisition Solution. Data were acquired on a Helios mass cytometer (Fluidigm) and normalized using the manufacturer’s bead-based normalization protocol.

Following normalization, initial cleanup of cell debris, removal of doublets, and exclusion of dead cells were performed using FlowJo software (v10.5.3). Cleaned FCS files were then analyzed using the R-based Cytofkit package (v3.8). For each sample, 10,000 events were randomly subsampled, and unsupervised clustering was performed using the Rphenograph algorithm incorporating all markers in the antibody panel. Dimensionality reduction was conducted using t-distributed stochastic neighbor embedding (t-SNE), and cluster identities were visualized on t-SNE maps. Relative marker expression and cluster abundances across treatment groups were visualized using heatmaps. CyTOF analyses were performed using five independent tumors per treatment group (n = 5), with each tumor considered an independent biological replicate.

### 2.3 *In vivo* mouse model

Female BALB/c mice aged 6–8 weeks were purchased from The Jackson Laboratory and housed under pathogen-free conditions in accordance with institutional animal care and use the Institutional Animal Care and Use (IACUC) committee guidelines. For bilateral tumor models, 0.4- and 0.2 million 4T1 cells suspended in 100 μL of PBS were injected subcutaneously into the right thigh (primary) and left flank (abscopal) on day 0 and day 2, respectively. Mice were randomized into treatment groups (N = 6 for control group, N = 7 mice for treatment group) once primary tumors reached ~50–100 mm³. RT was delivered to the primary tumor only using a Small Animal Radiation Research Platform (SARRP) at a dose of 8 Gy per fraction for one fraction, with shielding applied to protect surrounding tissue and the abscopal site. Mice were anesthetized with inhaled isoflurane during irradiation procedures. Fostamatinib (R788; MedChemExpress), a prodrug of the active metabolite R406, was used as the pharmacologic SYK-targeting agent in this study. R406 has been previously characterized as an ATP-competitive inhibitor of SYK, although direct R406 exposure and SYK target engagement were not measured under the experimental conditions used here (24, 25). Fostamatinib was administered intraperitoneally at 80 mg/kg daily, starting 1 day before the initiation of RT and continuing throughout the course of treatment. Anti-PD-1 antibody (clone RMP1-14, Bio X Cell) was administered intraperitoneally at 200 μg per dose every 3 days, beginning on the first day of RT. Tumor volumes were measured every 2–3 days using calipers, and calculated as (length×width^2^)/2. Investigators were aware of group allocation during treatment administration, as radiotherapy delivery and drug administration required knowledge of treatment assignment. Mice were euthanized at the experimental endpoint or predefined time points by gradual-fill carbon dioxide (CO_2_) inhalation using a displacement rate of 30–70% of the chamber volume per minute, followed by a secondary physical method to confirm death, in accordance with the recommendations of the American Veterinary Medical Association (AVMA) Guidelines for the Euthanasia of Animals. Abscopal tumors were harvested for downstream analyses, including flow cytometry, CyTOF, and RNA sequencing. All animal procedures were approved by the Mayo Clinic Institutional Animal Care and Use Committee (IACUC), Rochester, MN, USA, under protocol number A00003935-18-R24 and were conducted in accordance with institutional guidelines and the ARRIVE guidelines.

### 2.4 Public transcriptomic and single-cell analysis

Publicly available transcriptomic datasets were analyzed to assess *SYK* expression and its association with clinical and immunological features in breast cancer. *SYK* mRNA expression across normal and tumor tissues was evaluated using the TIMER 2.0 database (http://timer.cistrome.org/), which aggregates normalized RNA-seq data from GTEx and TCGA cohorts. Subtype-specific *SYK* expression patterns and survival analyses were performed using the METABRIC breast cancer dataset, accessed via cBioPortal (26). The Meta-TNBC cohort was assembled by integrating gene-expression and clinical data from TCGA-BRCA, METABRIC, E-TABM-158, NKI, GSE103091, GSE20486, GSE24450, GSE7390, GSE1456, GSE19783, and GSE16446. A total of 3,980 patients with breast cancer were initially evaluated. TNBC was defined as estrogen receptor (ER) and progesterone receptor (PR) expression <1% by immunohistochemistry and HER2 negativity (IHC 0-1+ or IHC 2+ with negative *in situ* hybridization), where receptor information was available. Patients with non-TNBC (n=3,211) or unavailable survival information (n=177) were excluded, resulting in 592 patients with TNBC (27). For the 10-year overall survival analysis, follow-up was administratively censored at 10 years. After restricting the analysis to patients with complete *SYK* expression and survival data meeting the prespecified analysis criteria, 468 patients were included in the final survival analysis. Individual datasets were processed separately before integration, and batch effects across datasets were adjusted using the R limma package. Immune cell composition was estimated using TIMER2.0-based immune deconvolution (28–30). Detailed information regarding the individual datasets, including platform, sample size, and availability of treatment and radiotherapy information, is provided in **Supplementary Table 1**. For survival analyses, the optimal cutoff for *SYK* expression was determined using maximally selected rank statistics implemented in the R survminer package, and patients were subsequently classified into *SYK*-high and *SYK*-low groups.

For single-cell RNA-seq analysis, we utilized the breast cancer dataset (SCP1106) to characterize *SYK* expression across tumor-infiltrating stromal and immune populations in TNBC (31), and the normal tissue dataset (SCP1731) to examine *SYK* expression in normal breast tissue (32). Processed data were accessed via the Broad Institute Single Cell Portal (https://singlecell.broadinstitute.org/single_cell) visualization interface and expression patterns were interpreted using annotated t-SNE projections and cell type-specific distribution plots. These complementary bulk and single-cell analyses enabled a comprehensive assessment of *SYK* expression dynamics in breast cancer tissues.

### 2.5 Clonogenic assay

Clonogenic survival assays were performed to assess radiosensitivity and response to combination treatment *in vitro*. Breast cancer cells (MDA-MB-468 and 4T1) were seeded at appropriate density (0.5K-20K/well) into 6-well plates and allowed to attach overnight. Cells were pretreated with the SYKi or vehicle control for 2 hours prior to irradiation. Ionizing radiation was delivered at indicated dose using an X-ray irradiator (Faxitron CellRad or equivalent). For combination treatments, olaparib (Selleckchem) or cisplatin (Sigma-Aldrich) was added concurrently with SYKi as indicated. After 10–14 days, colonies were fixed with 4% paraformaldehyde, stained with 0.5% crystal violet, and counted using ImageJ (v1.54). Colonies containing ≥50 cells were defined as survivors. Surviving fractions were normalized to the plating efficiency of the non-irradiated control group. Statistical comparisons were made using two-way ANOVA with multiple comparisons. Clonogenic assays were performed in three independent biological experiments (n = 3), with technical replicate wells within each experiment; independently cultured experiments served as the experimental unit.

### 2.6 Calcein release assay

Calcein release assays were performed to assess peripheral blood mononuclear cells (PBMCs)-mediated tumor cell cytotoxicity in an tumor cells/PBMC co-culture system. PBMCs were isolated from de-identified leukocyte reduction system (LRS) cones obtained from healthy blood donors as described below and activated with anti-CD3/CD28. MDA-MB-468 and HCC1806 cells were labeled with 10 μM Calcein-AM (Thermo Fisher Scientific) in serum-free medium at 37 °C for 30 minutes, followed by two washes with complete medium. Labeled tumor cells were seeded into 96-well U-bottom plates at 1 × 10^4^ cells/well and co-cultured with activated PBMCs at the indicated effector-to-target (E:T) ratios, with or without SYKi and anti-PD-1 antibody as indicated, for 4 hours at 37 °C. Following incubation, supernatants were collected and calcein fluorescence was measured using a microplate reader (excitation/emission: 485/520 nm). Maximum release was determined by treatment of target cells with 1% Triton X-100, and spontaneous release was measured from target cells cultured alone. Specific lysis was calculated as: % lysis = [(experimental release − spontaneous release)/(maximum release − spontaneous release)] × 100. Experiments were performed using PBMCs from three independent healthy donors (n = 3), with each donor considered an independent biological replicate.

### 2.7 Flow cytometry

PBMCs were isolated from de-identified leukocyte reduction system (LRS) cones obtained from healthy blood donors through an institutional service at Mayo Clinic. The specimens were provided without donor identifiers, and the investigators had no access to donor-identifying information. PBMCs were isolated from healthy donor whole blood using the Human PBMC Isolation Kit (STEMCELL Technologies) according to the manufacturer’s instructions. Isolated PBMCs were washed twice with PBS and cultured in RPMI-1640 medium. Cells were treated with the SYKi at the indicated concentrations for 72 hours. Following treatment, cells were collected using non-enzymatic dissociation buffer, washed twice with PBS, and resuspended in FACS buffer (PBS containing 2% FBS). Fc receptors were blocked with human TruStain FcX (BioLegend), and cells were stained with fluorochrome-conjugated antibodies against CD11b, CD206, and CD80 (BioLegend) for 30 minutes at 4 °C in the dark. Live/dead discrimination was performed using Zombie Aqua viability dye. After staining, cells were washed, fixed with 1% paraformaldehyde, and analyzed on a BD LSRFortessa flow cytometer. Data was processed using FlowJo (v10). CD11b+ myeloid/monocytic populations were characterized based on surface expression of CD80 and CD206. CD11b+CD80+ and CD11b+CD206+ cells were quantified as CD80+ and CD206+ myeloid/monocytic populations, respectively. No monocyte isolation or ex vivo macrophage differentiation was performed. PBMC experiments were performed using cells from three independent healthy donors (n = 3), with each donor considered an independent biological replicate.

### 2.8 Cell culture

Human triple-negative breast cancer cell lines MDA-MB-468, HCC1806, and mouse 4T1 breast cancer cells were obtained from ATCC and cultured in DMEM or RPMI-1640 supplemented with 10% fetal bovine serum (FBS). All cells were maintained under humidified normoxic conditions at 37 °C with 5% CO_2_ and were routinely tested for Mycoplasma contamination every 2–3 months.

### 2.9 Statistical analysis

All statistical analyses were performed using R (v4.3.0) and GraphPad Prism (v10.0). Unless otherwise indicated, all tests were two-tailed, and statistical significance was defined as *P* < 0.05. Data are presented as mean ± SEM, with error bars in all figures representing SEM. Representative experiments were independently repeated at least three times unless otherwise noted. Comparisons between two groups were performed using unpaired two-tailed Student’s t-tests or Wilcoxon rank-sum tests, as indicated in the corresponding figure legends. Comparisons involving multiple groups were analyzed using one-way or two-way ANOVA with appropriate *post hoc* multiple-comparison tests, unless otherwise specified. For exploratory immune-profiling analyses involving prespecified pairwise comparisons, unadjusted p-values were reported and interpreted cautiously. Tumor growth curves were analyzed using two-way ANOVA with repeated measures. Overall survival was administratively censored at 10 years. Survival outcomes were assessed by the Kaplan-Meier method with log-rank tests. Hazard ratios (HRs) and corresponding 95% confidence intervals (CIs) were estimated using univariable Cox proportional hazards regression. Correlation analyses were performed using Spearman’s rank correlation. For bulk and single-cell transcriptomic analyses, differential expression was assessed using model-based approaches with multiple-testing correction applied using the Benjamini-Hochberg false discovery rate. Biological replicates represent independent samples derived from distinct biological entities (e.g., independent cell cultures or individual animals), whereas technical replicates reflect repeated measurements of the same sample; all statistical analyses were conducted on biological replicates unless otherwise specified.

## 3 Results

### 3.1 SYK inhibition sensitizes breast cancer to radiotherapy

We examined *SYK* expression across human malignancies using pan-cancer transcriptomic datasets (33). *SYK* was significantly upregulated in multiple tumor types relative to matched normal tissues, including breast, colon, liver, and lung cancers (**Supplementary Figure 1A**). In breast cancer specifically, *SYK* mRNA levels were markedly elevated in tumor specimens compared to normal breast tissue (p<0.0001; **Figure 1A**), with the highest expression observed in the basal-like subtype which is enriched for TNBC (**Figure 1B**). We next evaluated the association between *SYK* expression and TNBC prognosis using a pooled cohort of publicly available data sets with survival outcomes that we had previously assembled (Meta-TNBC) (27). High *SYK* expression was significantly associated with inferior survival (HR = 2.65, 95% CI 1.73-4.06; log-rank p<0.0001; **Figure 1C**). In the METABRIC dataset (26), among radiotherapy-treated patients specifically, high *SYK* expression was associated with significantly worse outcomes compared to those with low *SYK* expression (HR = 1.34, 95% CI 1.05-1.69; log-rank p=0.016; **Figure 1D**).

**Figure 1:**
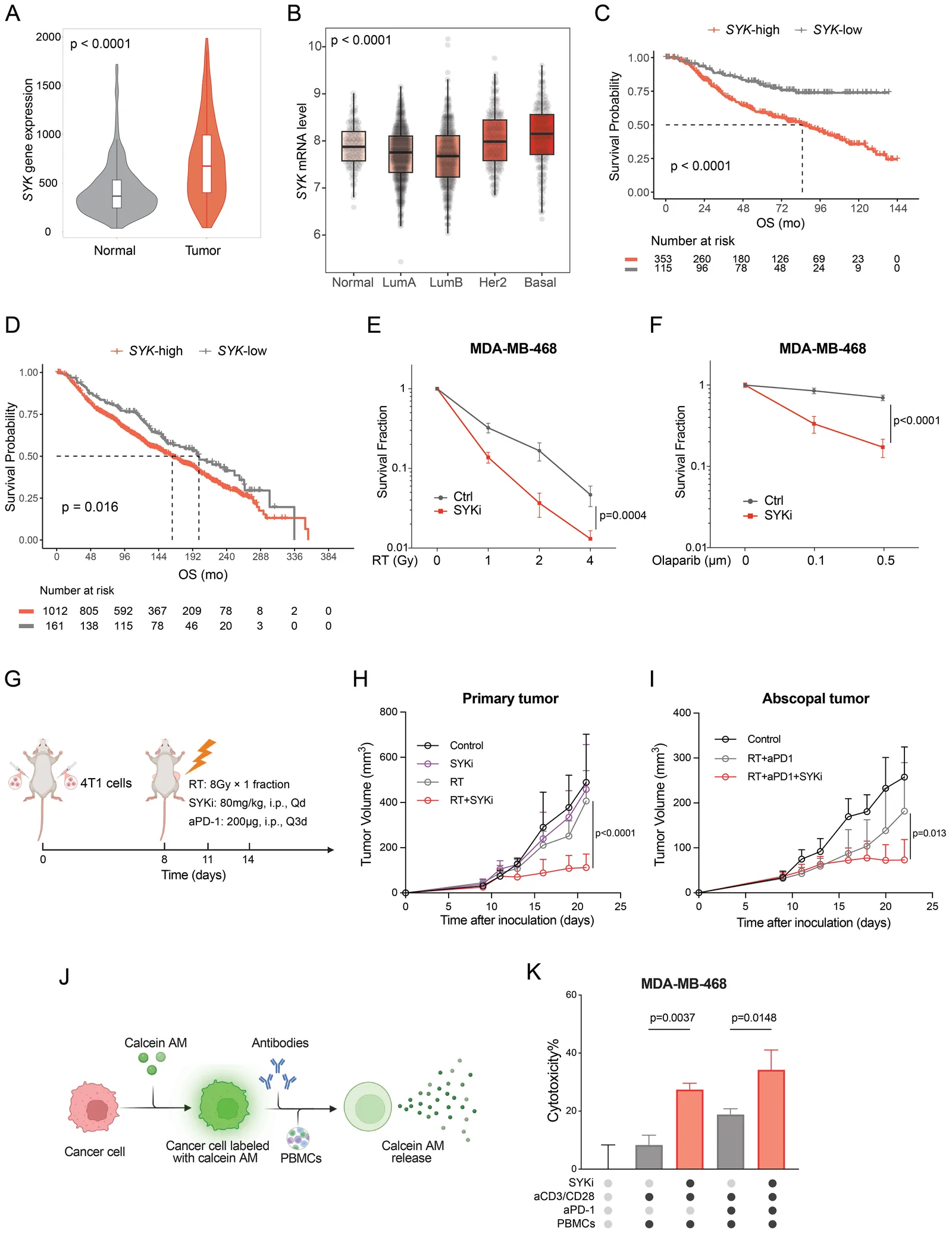
*SYK* is upregulated in breast cancer and its inhibition sensitizes tumors to radiotherapy. **(A)** Violin plot of *SYK* mRNA expression in normal versus breast tumor tissues (TNMplot database); significance was assessed by two-sided Wilcoxon rank-sum test. **(B)** Boxplots showing *SYK* expression across molecular subtypes of breast cancer, with the highest expression observed in basal-like breast cancer; comparisons performed by Kruskal-Wallis test followed by *post hoc* pairwise Wilcoxon tests. **(C)** Kaplan-Meier analysis of overall survival (OS) in a TNBC meta-cohort (n=468), showing inferior outcomes in patients with *SYK* high expression; significance determined by log-rank test. **(D)** Kaplan-Meier analysis of OS in the METABRIC breast cancer cohort (n=1,173) treated with radiotherapy, demonstrating poorer survival in *SYK*-high tumors; significance determined by log-rank test. Patients were stratified into SYK-high and SYK-low groups using an optimized expression cutoff. **(E, F)** Clonogenic survival assays in MDA-MB-468 treated with radiotherapy **(E)** and olaparib **(F)** in the presence or absence of the SYK inhibitor (SYKi) (fostamatinib), showing enhanced cytotoxicity with SYK inhibition. Representative crystal violet-stained colony images are shown below each survival curve. Data represent mean ± SEM from three independent biological experiments (n = 3) with statistical significance assessed by two-way ANOVA with multiple comparisons. **(G)** Schematic of *in vivo* experimental design: 4T1 cells were implanted bilaterally into BALB/c mice, followed by treatment with RT (8 Gy × 1 fraction), SYKi (80 mg/kg, i.p., daily), and/or PD-1 blockade (200 μg, i.p., every 3 days). **(H, I)** Tumor growth curves of primary **(H)** and abscopal **(I)** tumors from two independent bilateral 4T1 tumor experiments. In **(H)**, mice were treated with vehicle control, SYKi (80 mg/kg i.p., daily), RT (8 Gy × 1), or RT + SYKi. SYKi significantly sensitized primary tumors to radiotherapy, resulting in reduced tumor growth compared to RT alone. In **(I)**, mice were treated with vehicle, RT + anti-PD-1 (aPD-1), or RT + aPD-1 + SYKi. The addition of SYKi significantly enhanced abscopal tumor control relative to RT + aPD-1. Data are presented as mean ± SEM. Statistical significance was assessed using two-way ANOVA with multiple comparisons. **(J)** Schematic of the calcein release assay used to assess PBMC-mediated tumor cell killing in the presence or absence of SYKi and immune stimulation. **(K)** Calcein release assay in MDA-MB-468 cells demonstrating that SYK inhibition enhanced PBMC-mediated cytotoxicity following CD3/CD28 stimulation, with further enhancement in the presence of PD-1 blockade. Data represent mean ± SEM from three independent healthy donors (n = 3); statistical significance assessed by Student’s t-test.

We have previously reported that high tumor-intrinsic *SYK* expression promotes resistance to DNA damaging therapy by enhancing DNA double-stand break resection and homologous recombination (HR)-mediated DNA repair (34). Consistently, SYK inhibition with fostamatinib significantly sensitized SYK-expressing MDA-MB-468 human and 4T1 mouse TNBC cells to ionizing radiation (**Figure 1E**, **Supplementary Figure 1B**). Additionally, the sensitizing effect of fostamatinib extended beyond radiotherapy to other DNA damaging treatments such as olaparib (**Figure 1F**, **Supplementary Figure 1C**).

Given the role of SYK in immune signaling and the clinical relevance of PD-1 blockade in TNBC, we next investigated whether SYK inhibition could enhance the response to radiotherapy in an immunocompetent setting and potentiate the efficacy of PD-1 blockade. We therefore employed a bilateral 4T1 syngeneic abscopal tumor model (**Figure 1G**). RT combined with SYKi significantly suppressed primary tumor growth relative to RT alone (**Figure 1H**). The enhanced efficacy compared favorably with the radiosensitizing effect of SYK blockade in 4T1 cells observed *in vitro* (**Supplementary Figure 1B**), suggesting an additional systemic effect. However, the addition of SYK inhibition to radiotherapy failed to elicit an effective systemic antitumor immune response at distant abscopal tumors (**Supplementary Figure 1D**). In contrast, the addition of SYK inhibition to RT+aPD-1 significantly enhanced tumor control at the distant non-irradiated site compared with RT+aPD-1 alone (**Figure 1I**). Primary tumor control was not further enhanced by the addition of aPD-1, likely due to the short timeline of the experiment with abscopal tumors in the control and RT+aPD-1 arms reaching humane endpoints prior to the emergence of a statistically significant difference at the primary tumors (**Supplementary Figure 1E**). Collectively, these results suggest that SYK inhibition enhances the radiosensitivity of TNBC and stimulates systemic antitumor immune responses when combined with PD-1 blockade.

To further study the effects of SYK inhibition on immune effector function we employed ex vivo calcein release assays using CD3/CD28 activated human peripheral blood mononuclear cells (PBMCs) from healthy donors (**Figure 1J**). SYK inhibition potentiated PBMC-mediated cytotoxicity in MDA-MB-468 cells, particularly when combined with aPD-1 (**Figure 1K**). SYK inhibition also enhanced cytotoxicity in another breast cancer cell line HCC1806 cell (**Supplementary Figure 1F**). We concluded that SYK inhibition may strengthen anti-tumor immune-effector function.

### 3.2 SYK drives immune dysregulation in TNBC

Given the poor outcomes observed in *SYK* expressing TNBC, and that SYK inhibition could enhance immune mediated tumor cell killing, we hypothesized that SYK may drive immune dysregulation within the tumor microenvironment.

To test this hypothesis, we first defined the molecular profiles associated with *SYK* expression by performing differential expression analysis in low and high *SYK* expressing tumors amongst radiotherapy-treated tumors in the METABRIC dataset. *SYK*-high breast cancers demonstrated differential expression of genes enriched for a number of immune and inflammatory pathways (**Figures 2A, B**). Of note, enrichment analysis of upregulated genes in the METABRIC dataset revealed significant activation of NF-κB signaling and pathways linked to autoinflammatory disease (**Supplementary Figure 2A**).

**Figure 2:**
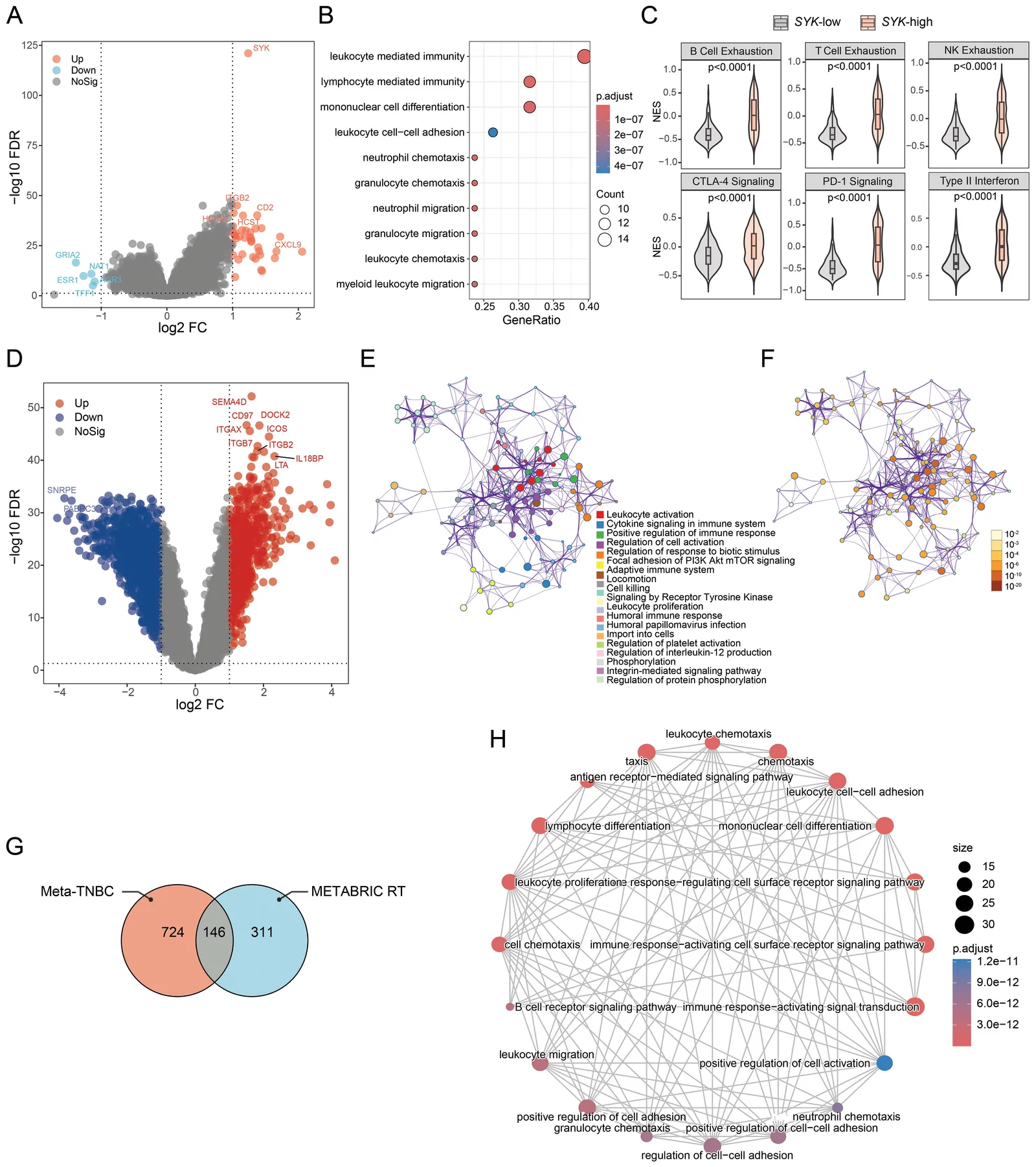
Immune dysregulation in *SYK*-high TNBC and potentiated effects of SYK inhibition with immunotherapy. **(A)** Volcano plot of differentially expressed genes (DEGs) between *SYK*-high and *SYK*-low tumors in the METABRIC breast cancer cohort treated with radiotherapy, showing immune-related genes enriched in the *SYK*-high group. **(B)** Gene ontology (GO) enrichment analysis of upregulated DEGs in *SYK*-high tumors from the METABRIC radiotherapy cohort, indicating enrichment in extensive immune-related pathways. **(C)** Single-sample gene set enrichment analysis (ssGSEA) analysis comparing pathway activity between *SYK*-high and *SYK*-low tumors, demonstrating broad upregulation of immune-related pathways in *SYK*-high tumors, including T/B/NK cell exhaustion, CTLA-4 and PD-1 checkpoint signaling, and type II interferon signaling. Statistical significance determined by two-sided Wilcoxon rank-sum test. **(D)** Volcano plot of DEGs between *SYK*-high and *SYK*-low tumors in a TNBC meta-cohort, highlighting immune-related genes significantly upregulated in *SYK*-high tumors. **(E, F)** Enrichment network maps of upregulated DEGs in *SYK*-high tumors, demonstrating activation of immune and inflammatory pathways including leukocyte activation, cytokine signaling, and T cell receptor signaling. **(G)** Venn diagram showing the overlap of upregulated DEGs in *SYK*-high groups between the TNBC meta-cohort and the METABRIC radiotherapy-treated cohort. **(H)** Enrichment map of overlapping genes from **(G)**, highlighting immune and chemotaxis-related pathways.

Chronic activation of inflammatory pathways, including NF-κB and interferon signaling, has been shown to drive immune dysfunction and exhaustion by sustaining a proinflammatory environment that leads to T cell desensitization and reprograms macrophages toward dysfunctional and immunosuppressive states (35–38). Single-sample gene set enrichment analysis (ssGSEA) confirmed that *SYK*-high tumors exhibited features of extensive activation of inflammatory signaling together with immune exhaustion, including upregulation of B cell pathways, T cell and NK cell exhaustion programs, PD-1 and CTLA-4 checkpoint signaling, and type I/II interferon responses (**Figure 2C**; **Supplementary Figure 2B**). Consistently, in Meta-TNBC, *SYK* expression was associated with a number of immune-related processes, including cytokine signaling, leukocyte activation, and adaptive immune responses (**Figures 2D–F**). To further refine the molecular programs associated with *SYK* expression, we next focused on overlapping genes that were consistently upregulated in *SYK*-high tumors across both the Meta-TNBC cohort and METABRIC radiotherapy-treated subset. This analysis yielded 146 shared genes (**Figure 2G**), which were again enriched for inflammatory processes (**Figure 2H****).** Based on these collective results, we concluded that elevated *SYK* expression in TNBC may be linked with an immune-exhausted tumor microenvironment.

### 3.3 SYK inhibition augments systemic immune response to radiotherapy plus PD-1 blockade in abscopal tumors by activating the tumor microenvironment

Having observed that *SYK*-high tumors exhibit features of immune exhaustion, we next sought to determine how SYK inhibition alters the tumor immune landscape. To this end, we performed RNA-seq on non-irradiated 4T1 tumors distant from the local irradiated primary site (**Figure 1I**). Differential expression analysis demonstrated that compared with RT+aPD-1 alone, RT+aPD-1+SYKi upregulated pathways related to adaptive immune response, including lymphocyte activation and humoral effector responses (**Figures 3A, B**). Downregulated pathways were enriched for extracellular matrix organization and neutrophil-associated responses (**Figure 3C**). Immune deconvolution using the TIMER2.0 platform further revealed that abscopal tumors treated with RT+aPD-1+SYKi displayed a reduced inferred abundance of M2-like macrophages, with trends toward increased M1-like macrophages and an elevated M1/M2 ratio, without substantial alteration in overall macrophage abundance (**Figures 3D–F**, **Supplementary Figure 3A**). In addition, non-regulatory CD4^+^ T cells and endothelial cell abundance were reduced with the triple combination treatment (**Figures 3G, H**). No significant alterations were detected in other predominant immune subsets (**Supplementary Figures 3B–G**). We concluded that SYK inhibition was associated with reduced M2-like macrophage abundance and broader remodeling of the tumor immune microenvironment, which may contribute to the enhanced systemic antitumor response following RT+aPD-1.

**Figure 3:**
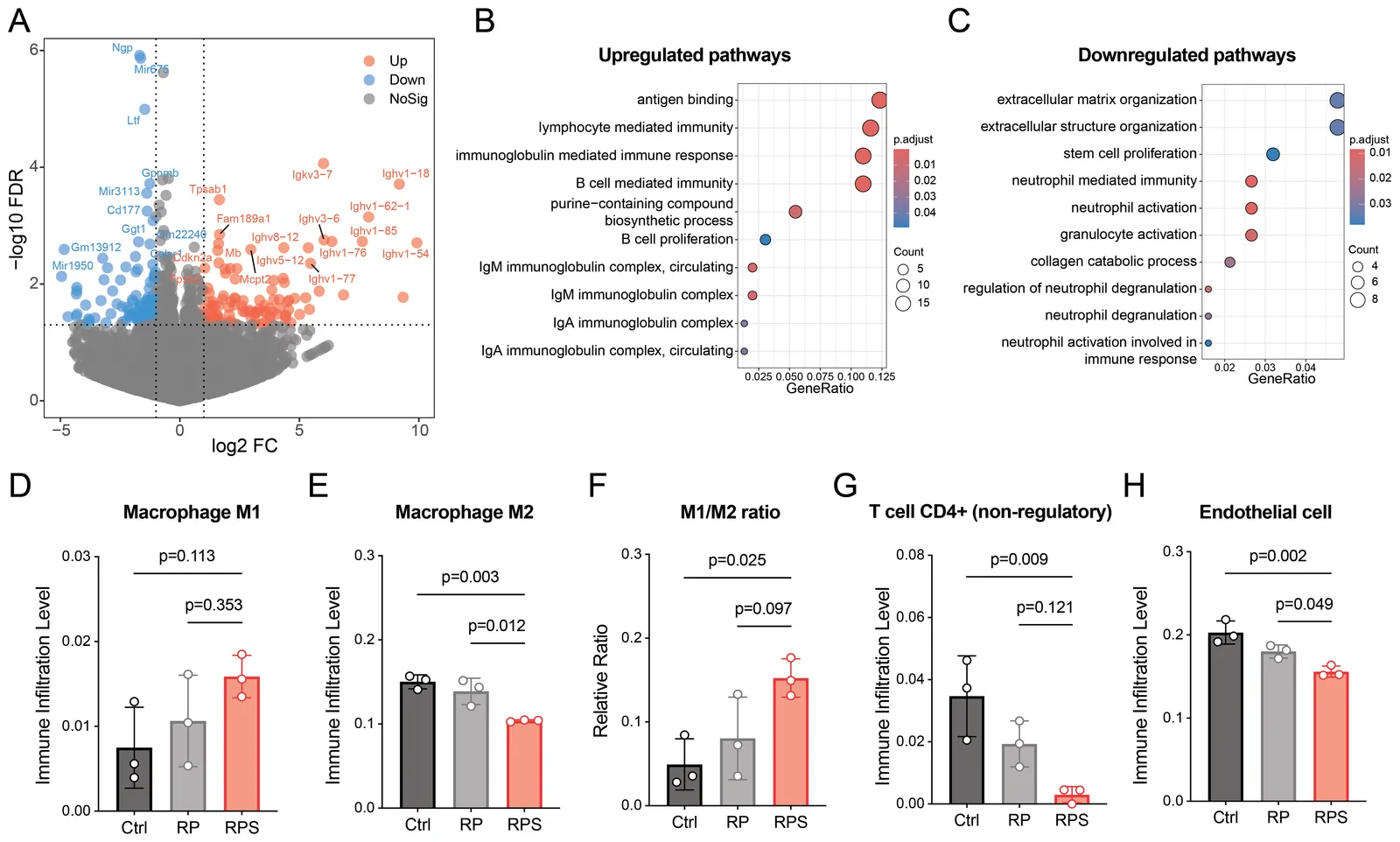
SYK inhibition enhances the abscopal efficacy of radiotherapy and PD-1 blockade through immune remodeling. **(A)** Volcano plot of DEGs in abscopal tumors comparing RT+aPD-1+SYKi (RPS) versus RT+aPD-1 (RP), highlighting upregulated and downregulated genes. **(B, C)** Gene ontology enrichment analysis of upregulated **(B)** and downregulated **(C)** DEGs showing enhanced lymphocyte- and antibody-mediated immune responses, alongside reduced extracellular matrix organization and neutrophil-related pathways. **(D-F)** TIMER2.0-based immune deconvolution estimates showing reduced M2 macrophages and trends toward higher M1-like macrophage abundance and M1/M2-like ratio in RPS-treated abscopal tumors compared with RP and control groups. Pairwise comparisons were performed using two-sided Student’s t-tests, with unadjusted p-values reported for these exploratory analyses. **(G, H)** TIMER2.0-based immune deconvolution showing decreased infiltration of non-regulatory CD4^+^ T cells **(G)** and endothelial cells **(H)** in RPS-treated tumors.

To corroborate the immune changes identified by RNA-seq, we performed high-dimensional CyTOF profiling of abscopal tumors collected at days 7 and 14 after irradiation (**Figures 4A, B**). The complete CyTOF antibody panel, including antibody clones and metal tags, is provided in **Supplementary Table 2**, with marker-expression profiles and cluster annotations shown in **Supplementary Figure 3**. SYK inhibition combined with RT + aPD-1 therapy was associated with significant changes in the tumor immune microenvironment (**Figures 4C–E**, **Supplementary Figures 5A–C**). At day 7 post-RT, treatment-associated changes were observed across multiple myeloid populations, including an increase in the iNOS-high M1-like myeloid population (C12) and a lower abundance of the CD206+/CD163-high M2-like myeloid population (C24) in RPS-treated tumors compared with the RP group (**Supplementary Figures 5D, E**). Changes were also observed across several neutrophil populations, including neutrophils (C3), neutrophil-like populations (C8 and C19), and proliferating neutrophils (C14), as well as an activated APC/myeloid population (C15) (**Supplementary Figures 5F–K**). By day 14, broader remodeling of the myeloid compartment was observed, including significant changes in a Ly6C-high myeloid population (C18), monocyte/M-MDSC-like cells (C11), and an activated APC/myeloid population (C15) (**Figures 4F–H**). In contrast, the F4/80+ macrophage population (C20) did not differ significantly among treatment groups (**Figure 4I**), while regulatory T cells (C16) also showed treatment-associated changes (**Figure 4J**). Together, these findings demonstrate temporally distinct immune remodeling, characterized by early changes in macrophage and neutrophil populations followed by broader remodeling of monocytic and other myeloid populations at day 14.

**Figure 4:**
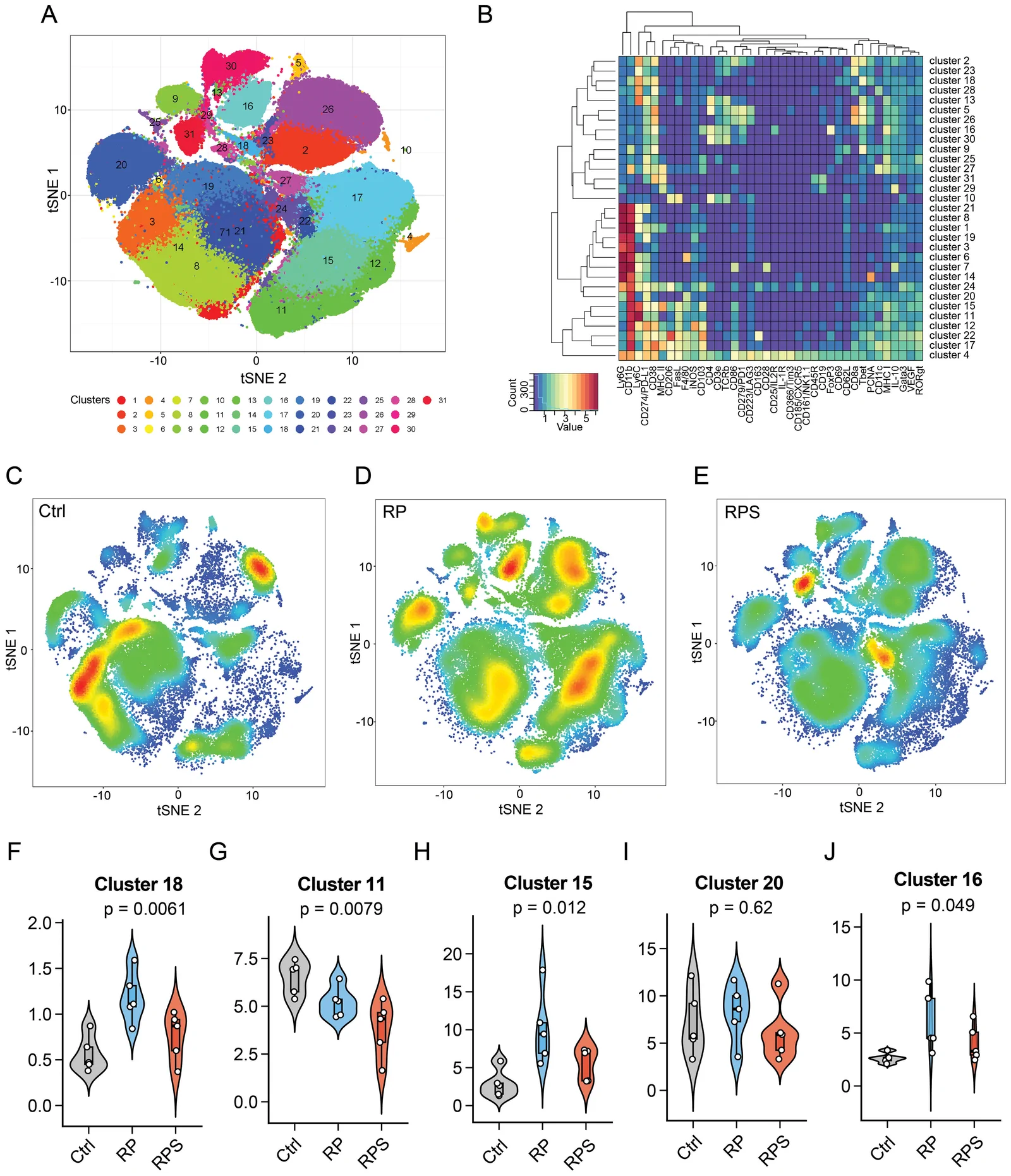
SYK inhibition is associated with myeloid immune remodeling in the abscopal tumor microenvironment. **(A)** t-SNE plot of CyTOF data from abscopal tumors across treatment groups, showing 31 identified immune cell clusters. **(B)** Heatmap showing relative expression patterns of CyTOF markers across the 31 clusters. **(C–E)** Representative t-SNE density plots showing immune cell distributions in abscopal tumors at day 14 post-treatment from control (Ctrl) **(C)**, RT + anti-PD-1 (RP) **(D)**, and RT + anti-PD-1 + SYKi (RPS) **(E)** groups. **(F–J)** Relative abundance of representative immune cell populations, including C18 (Ly6C-high myeloid population) **(F)**, C11 (monocyte/M-MDSC-like population) **(G)**, C15 (activated APC/myeloid population) **(H)**, C20 (F4/80+ macrophages) **(I)**, and C16 (regulatory T cells) **(J)**. Individual points represent independent tumors (n = 5 per group). P values indicate overall differences among the three treatment groups and were determined by one-way ANOVA.

Given our observations that SYK inhibition alters macrophage polarization, most notably by reducing M2-like macrophages, we next examined the cellular and transcriptional context of *SYK* expression. Single-cell RNA-seq analysis of human breast cancer specimens revealed that *SYK* expression is predominantly enriched within myeloid populations, whereas expression in normal breast tissue remains comparatively low (**Figures 5A, B****;** **Supplementary Figures 6A, B**). In the Meta-TNBC dataset, TIMER2.0-based immune deconvolution revealed that tumors with high *SYK* expression were characterized by an increased abundance of macrophages (**Figure 5C**), consistent with an association between *SYK* expression and a macrophage-enriched tumor immune landscape. Pathway enrichment analysis further demonstrated that *SYK*-high tumors exhibited transcriptional signatures associated with monocyte differentiation and polarization (**Figure 5D**). To further examine the effects of SYK inhibition on human myeloid/monocytic cells, we performed flow cytometry on PBMCs derived from healthy donors. SYK inhibition increased the proportion of CD11b+CD80+ cells while decreasing the CD11b+CD206+ population, resulting in an increased CD80+/CD206+ ratio (**Figures 5E–H**, **Supplementary Figure 6C**).

**Figure 5:**
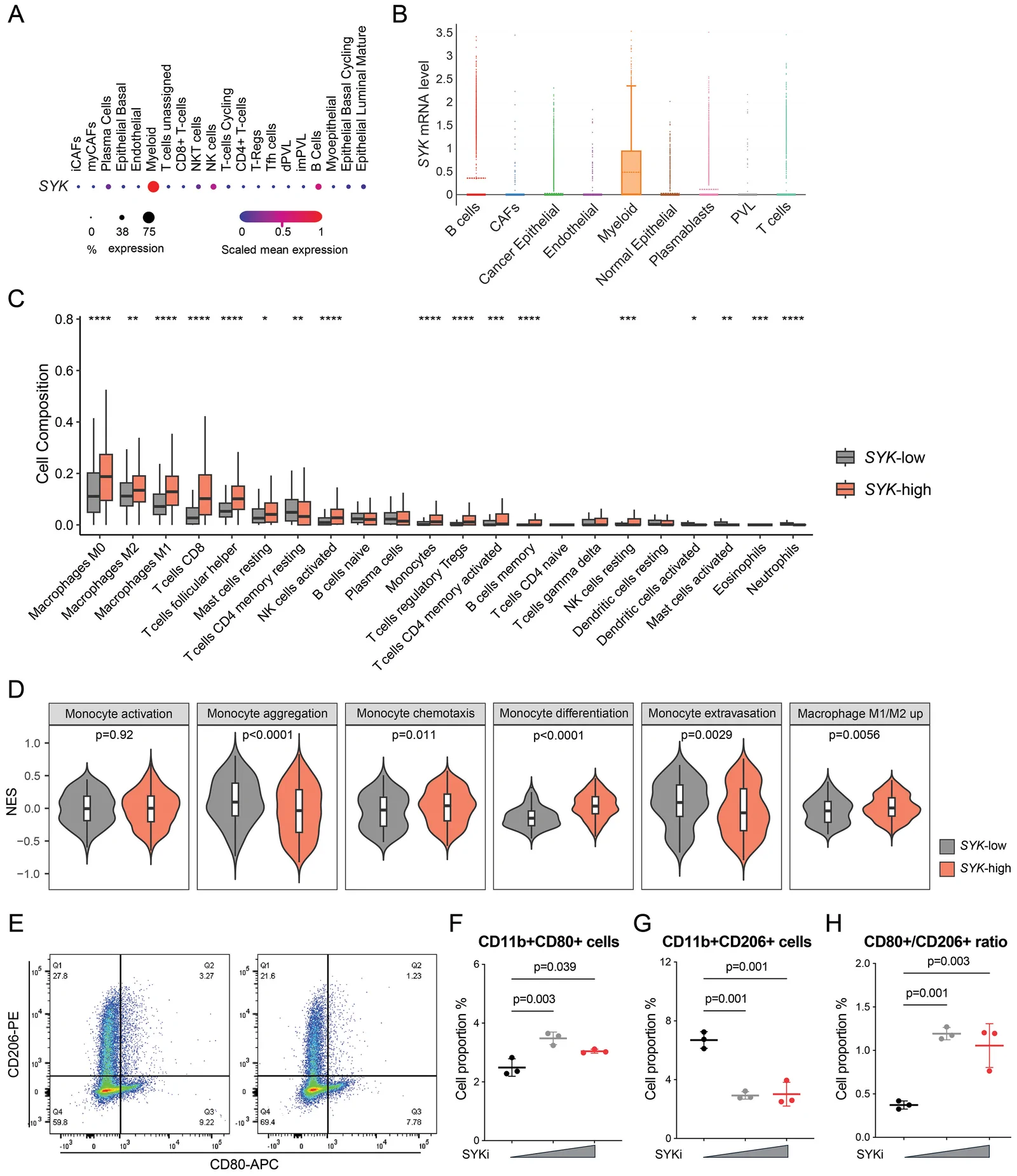
Macrophage-associated and myeloid immune remodeling following SYK inhibition in TNBC. **(A)** Single-cell RNA-seq (scRNA-seq) analysis of breast cancer showing *SYK* expression across major cell populations, with predominant enrichment in myeloid cells. **(B)** *SYK* mRNA expression across cell lineages in the single-cell RNA-seq dataset, confirming highest levels in myeloid cells. **(C)** TIMER2.0-based immune deconvolution of a TNBC meta-cohort comparing *SYK*-high and SYK-low tumors, revealing enrichment of macrophage populations in *SYK*-high tumors. **(D)** ssGSEA showing monocyte-related pathways (differentiation and M1/M2 polarization) enriched in *SYK*-high tumors. **(E)** Representative gating strategy for flow cytometric analysis of healthy-donor PBMCs, showing identification of CD11b+CD80+ and CD11b+CD206+ myeloid/monocytic populations. **(F–H)** Flow cytometric quantification showing that fostamatinib treatment increased the CD11b+CD80+ population **(F)**, decreased the CD11b+CD206+ population **(G)**, and increased the CD80+/CD206+ ratio **(H)** in a dose-dependent manner. Black bars indicate vehicle control (0 μM), grey bars indicate SYKi at 2 μM, and red bars indicate SYKi at 4 μM. For panels **(B-D)**, comparisons between *SYK*-high and *SYK*-low groups were performed using the two-sided Wilcoxon rank-sum test, with unadjusted p-values reported for these exploratory analyses. For panels **(F-H)**, data represent mean ± SEM from three independent healthy donors (n = 3). Pairwise comparisons were performed using two-sided Student’s t-tests, with unadjusted p-values reported.

## 4 Discussion

This study uncovers a novel role for SYK in TNBC, linking its expression to both therapeutic resistance and immune dysfunction. Although SYK has been studied extensively in hematologic malignancies and immune signaling, its impact on radiotherapy response and the tumor immune microenvironment has remained poorly understood. Our results suggest that SYK contributes to therapeutic resistance and is associated with an immunosuppressive macrophage-dominant tumor landscape. Importantly, pharmacologic inhibition of SYK using fostamatinib sensitized tumors to DNA-damaging therapy and remodeled the tumor immune microenvironment, with prominent macrophage reprogramming, while enhancing responses to radiotherapy combined with PD-1 blockade. These findings extend the biological scope of SYK beyond its canonical signaling roles and highlight its potential therapeutic relevance as a target for overcoming treatment resistance, particularly in combination with radiotherapy plus PD-1 blockade in patients with early-stage TNBC and residual disease after neoadjuvant systemic therapy (39, 40). In this setting, pembrolizumab combined with neoadjuvant chemotherapy followed by adjuvant pembrolizumab with radiotherapy as clinically indicated has become a standard-of-care systemic treatment based on the KEYNOTE-522 trial (41, 42).

A key contribution of this study is the identification of macrophage reprogramming as a prominent component of the immune remodeling associated with SYK inhibition. Transcriptomic analyses revealed that *SYK*-high tumors are enriched in macrophages and exhibit transcriptional programs associated with monocyte differentiation and polarization. Single-cell analyses further demonstrated preferential *SYK* expression within myeloid populations, although higher-resolution characterization of macrophage subsets and activation states will be important to define the cell-state-specific role of SYK. Consistent with these observations, analysis of healthy-donor PBMCs demonstrated that SYK inhibition decreased the CD11b+CD206+ myeloid/monocytic population while increasing CD11b+CD80+ cells, resulting in an increased CD80+/CD206+ ratio. Although these PBMC experiments do not establish macrophage differentiation or definitive M1/M2 polarization, they provide complementary evidence that SYK inhibition can alter the phenotype of human myeloid/monocytic cells. These findings are particularly relevant given the established role of M2-like TAMs as a barrier to both radiotherapy and immunotherapy efficacy, as they promote immune evasion, angiogenesis, and tumor progression (43). The reduction in M2-like macrophage abundance observed in the tumor immune profiling analyses may contribute to a more permissive immune context for T cell activation and response to checkpoint blockade. Consistent with these findings, transcriptomic analyses demonstrated enrichment of adaptive immune response and lymphocyte activation pathways, although direct functional characterization of tumor-infiltrating T cells was beyond the scope of the present study. The enhanced PBMC-mediated cytotoxicity observed ex vivo provides complementary proof-of-concept evidence for the immunomodulatory effects of SYK inhibition.

Although macrophage reprogramming was a prominent immune alteration associated with SYK inhibition, we also observed changes in neutrophil, monocytic/M-MDSC-like, APC/myeloid, and endothelial populations. CyTOF profiling revealed temporally distinct remodeling of the myeloid compartment. At day 7, treatment-associated changes were observed across several neutrophil populations together with iNOS-high M1-like and CD206+/CD163-high M2-like myeloid populations. By day 14, changes were observed in Ly6C-high myeloid, monocyte/M-MDSC-like, and activated APC/myeloid populations, whereas the F4/80+ macrophage population did not differ significantly among treatment groups. Together, these findings suggest that SYK inhibition induces broader and temporally heterogeneous restructuring of the myeloid compartment rather than acting exclusively through macrophages. From a translational perspective, our findings have several implications. The association between elevated *SYK* expression and poorer outcomes in TNBC provides clinical context for further investigation of SYK-targeted therapeutic strategies.

Our results extend our prior work linking SYK to homologous recombination repair and therapeutic resistance in TNBC (34). In that study we demonstrated that SYK enhanced homologous recombination by promoting DNA end resection and RAD51 recruitment, thereby increasing resistance to DNA-damaging therapies. Thus, SYK inhibition may simultaneously impair tumor-intrinsic DNA repair and remodel the tumor immune microenvironment, including by reprogramming SYK-enriched tumor-associated myeloid cells. This dual tumor-intrinsic and immune-modulatory activity provides a mechanistic rationale for combining SYK inhibition with radiotherapy and immune checkpoint blockade. Importantly, fostamatinib is an FDA-approved SYKi for chronic immune thrombocytopenia with an established safety profile (44). A Phase 1 trial of fostamatinib in combination with weekly paclitaxel is ongoing in patients with recurrent ovarian, fallopian tube, and peritoneal cancer (NCT03246074). Optimizing of dosing and scheduling will be required to maximize efficacy and minimize toxicity while leveraging both DNA repair and immune-mediated effects of combination therapy. Although fostamatinib (R788) is an established prodrug of the active SYK inhibitor R406, conversion to R406 and pharmacodynamic SYK target engagement were not directly measured under the experimental conditions used in the present study. Therefore, the pharmacologic findings should not be interpreted as definitive evidence that all observed treatment effects were exclusively SYK-dependent. In addition, R406 can inhibit other kinases, including FLT3 and LYN, which may have contributed to the observed effects. Future studies incorporating direct pharmacodynamic assessment of SYK signaling together with genetic SYK loss-of-function models will be important to establish target engagement and target specificity.

Although our integrated transcriptomic, CyTOF, and flow cytometric analyses consistently identified macrophage reprogramming as a prominent immune alteration following SYK inhibition, the present study was not designed to directly establish the relative contribution of macrophages to the therapeutic benefit. In addition, although fostamatinib is a clinically approved SYKi, future studies using genetic *SYK* loss-of-function models will be important to further establish target specificity and distinguish SYK-dependent effects from potential off-target kinase inhibition. Such studies, together with macrophage-specific depletion, functional characterization of macrophages, neutrophils, and MDSCs, cytokine profiling, and comprehensive analyses of tumor-infiltrating T-cell function, will further define how SYK inhibition coordinates innate and adaptive antitumor immunity. Finally, validation in independent human TNBC specimens using spatial or multiplex imaging approaches will be important to define the relationship between *SYK* expression and macrophage phenotypes within the human tumor microenvironment.

In summary, this study establishes SYK as a mediator of radioresistance and immune dysfunction in TNBC. Pharmacologic SYK inhibition remodels the tumor immune microenvironment, including through macrophage reprogramming, and enhances the efficacy of radiotherapy combined with PD-1 blockade. Our results provide a rationale for further investigation of SYKi-based combination strategies in early-stage and advanced TNBC.

## Data Availability

The original contributions presented in the study are included in the article/**Supplementary Material**. Further inquiries can be directed to the corresponding authors.
