# Supplementary material for: Targeting SYK enhances responses to radiotherapy and PD-1 blockade with myeloid immune remodeling in breast cancer
Source: Front Immunol. 2026 Sep 18;17:1929236. doi: 10.3389/fimmu.2026.1929236 (PMC13631016; doi:10.3389/fimmu.2026.1929236)
Supplement: Supplementary file 1 [file Table1.docx]

Supplementary Material

**Supplementary Figure 1.** **SYK is upregulated across multiple cancers and its inhibition enhances DNA-damaging therapy sensitivity**

**(A)** Pan-cancer analysis of *SYK* expression using the TIMER 2.0 database, showing significantly higher *SYK* mRNA levels in multiple tumor types compared with corresponding normal tissues; significance determined by two-sided Wilcoxon rank-sum test. **(B-C)** Clonogenic survival assays showing that SYKi potentiated the cytotoxic effects of radiotherapy (B) and olaparib (C) in 4T1 cells; significance assessed by two-way ANOVA. **(D-E)** Tumor growth curves from two independent bilateral 4T1 mouse experiments. (D) Abscopal tumor response in mice treated with control, SYKi, RT, or RT combined with SYKi. (E) Primary tumor response in mice treated with control, RT+anti-PD-1 (aPD-1), or RT+aPD-1+ SYKi. Data are presented as mean ± SEM. Statistical significance was assessed using two-way ANOVA with multiple comparisons. **(F)** Calcein release assays showing that SYKi enhanced CD3/CD28 stimulation and PBMC-mediated cytotoxicity against HCC1806. Data represent mean ± SEM; significance assessed by Student’s t-test. ACC, adrenocortical carcinoma; BLCA, bladder urothelial carcinoma; BRCA, breast invasive carcinoma (Basal, Her2, LumA, and LumB subtypes); CESC, cervical squamous cell carcinoma and endocervical adenocarcinoma; CHOL, cholangiocarcinoma; COAD, colon adenocarcinoma; DLBC, diffuse large B-cell lymphoma; ESCA, esophageal carcinoma; GBM, glioblastoma multiforme; HNSC, head and neck squamous cell carcinoma (HPV⁺/HPV⁻); KICH, kidney chromophobe; KIRC, kidney renal clear cell carcinoma; KIRP, kidney renal papillary cell carcinoma; LAML, acute myeloid leukemia; LGG, lower-grade glioma; LIHC, liver hepatocellular carcinoma; LUAD, lung adenocarcinoma; LUSC, lung squamous cell carcinoma; MESO, mesothelioma; OV, ovarian serous cystadenocarcinoma; PAAD, pancreatic adenocarcinoma; PCPG, pheochromocytoma and paraganglioma; PRAD, prostate adenocarcinoma; READ, rectum adenocarcinoma; SARC, sarcoma; SKCM, skin cutaneous melanoma; STAD, stomach adenocarcinoma; TGCT, testicular germ cell tumor; THCA, thyroid carcinoma; THYM, thymoma; UCEC, uterine corpus endometrial carcinoma; UCS, uterine carcinosarcoma; and UVM, uveal melanoma.

**Supplementary Figure 2. Validation of immune dysregulation associated with high SYK expression**

**(A)** GO enrichment analysis of upregulated genes in *SYK*-high tumors from the METABRIC radiotherapy-treated cohort, showing enrichment of immune-related pathways including NF-κB signaling and autoinflammatory disease signatures. **(B)** ssGSEA pathway analysis comparing *SYK*-high and *SYK*-low tumors, confirming broad upregulation of immune and inflammatory pathways in *SYK*-high tumors, including antigen presentation, apoptosis, cytotoxicity, IL-1/IL-6/IL-10 signaling, JAK-STAT signaling, NF-κB signaling, and immune checkpoint signaling; statistical significance determined by two-sided Wilcoxon rank-sum test.

**Supplementary Figure 3. Abscopal tumors treated with SYK inhibition show macrophage-specific remodeling without broad immune alterations**

**(A-G)** TIMER2.0-based estimates of immune infiltration in abscopal tumors. No significant changes were observed in total macrophages (A), regulatory T cells (Tregs) (B), CD8⁺ T cells (C), B cells (D), neutrophils (E), myeloid dendritic cells (F), or NK cells (G) across treatment groups. Data represent mean ± SEM; statistical significance determined by two-sided Student’s t-test.

**Supplementary Figure 4. Marker-expression profiles of the CyTOF panel**

Marker-specific t-SNE density plots showing the expression distribution of individual lineage, activation, and functional markers across tumor-infiltrating immune cells. The complete CyTOF antibody panel, including antibody clones and metal tags, is provided in Supplementary Table 2.

**Supplementary Figure 5. CyTOF profiling reveals myeloid immune remodeling following combined radiotherapy, PD-1 blockade, and SYK inhibition**
(A-C) t-SNE density plots of CyTOF analysis showing immune cell distributions in abscopal tumors from control (Ctrl), RT + anti-PD-1 (RP), and RT + anti-PD-1 + SYKi (RPS) groups at day 7 post-treatment. (D-K) Relative abundance of representative immune cell clusters, including C12 (iNOS-high M1-like myeloid population) (D), C24 (CD206+/CD163-high M2-like myeloid population) (E), C3 (neutrophils) (F), C14 (proliferating neutrophils) (G), C8 (neutrophil-like cells) (H), C19 (neutrophil-like cells) (I), C2 (activated CD8 T cells) (J), and C15 (activated APC/myeloid cells) (K). Individual points represent independent tumors (n = 5 per group). P values indicate overall differences among the three treatment groups and were calculated using one-way ANOVA.

**Supplementary Figure 6. SYK expression and myeloid/monocytic phenotypic changes following SYK inhibition**

**(A-B)** scRNA-seq analysis of normal breast tissue showing minimal *SYK* expression across stromal and immune populations, in contrast to tumor-associated enrichment (Figure 5A-B). **(C)** Flow cytometry gating strategy on healthy donor’s PBMCs for CD11b⁺CD80⁺ and CD11b⁺CD206⁺ myeloid/monocytic populations.
